# Association of lipoprotein lipase genetic risk score with cardiometabolic risk indicators in a healthy Qatari population using the Qatar Biobank data

**DOI:** 10.1371/journal.pone.0341641

**Published:** 2026-02-05

**Authors:** Maria M. AlAnazi, Julie A. Lovegrove, Karani Santhanakrishnan Vimaleswaran

**Affiliations:** 1 Hugh Sinclair Unit of Human Nutrition, Department of Food and Nutritional Sciences, University of Reading, Reading, United Kingdom; 2 Department of Nutrition Sciences, College of Health Sciences, Qatar University, Doha, Qatar; 3 Institute for Food, Nutrition and Health (IFNH), University of Reading, Reading, United Kingdom; Midwestern University, UNITED STATES OF AMERICA

## Abstract

Genetic variations within the Lipoprotein Lipase (*LPL)* gene have been shown to influence the risk of cardiometabolic diseases. However, their associations with cardiometabolic disease-related markers remain underexplored in Arab Qatari populations. Hence, we examined the association between a genetic risk score (GRS) based on three *LPL* single nucleotide polymorphisms (SNPs) and cardiometabolic indicators in a healthy Qatari population. A cross-sectional genetic association study was conducted using data from the Qatar Biobank population-based cohort, involving a sample of metabolically healthy Qatari adults (n = 6,919). The *LPL-*GRS was computed as the unweighted sum of risk alleles from three *LPL* SNPs: rs295 (C/A), rs301 (C/T), and rs320 (G/T). Associations between the GRS and metabolic markers were assessed using a generalized linear model, adjusting for age, sex, and body mass index. Individuals with high GRS (>5 risk alleles) showed a significant association with lower fat-free mass index values (β = −0.064, p = 0.029). In addition, a positive association was observed between GRS and fasting insulin levels (β = 0.035, p = 0.016). In addition, high GRS was significantly associated with lower high-density lipoprotein cholesterol (β = −0.025, p = 0.001) and higher triacylglycerol concentrations (β = 0.027, p = 0.0003) and systolic blood pressure (β = 0.007, p = 0.002), respectively. Our study shows that the *LPL*-GRS is associated with key cardiometabolic risk factors in this self-reported healthy Qatari population. These findings highlight the need for additional research to replicate these findings in independent and ethnically diverse cohorts, as well as the use of longitudinal studies to evaluate the predictive value of the GRS for future metabolic outcomes.

## Introduction

Cardiometabolic diseases are a group of interconnected conditions that include cardiovascular diseases and metabolic disorders such as type 2 diabetes (T2D) and obesity [1,2]. They share underlying mechanisms like chronic inflammation, endothelial dysfunction, impaired glucose and lipid metabolism, increased oxidative stress, and ectopic fat deposition [3–6]. Notably, obesity is the key modifiable risk factor that predisposes individuals to a range of metabolic and cardiovascular complications, including insulin resistance and T2D [7,8], dyslipidemia [9], hypertension [10], and cardiovascular diseases (CVDs) [11,12]. Remarkably, the prevalence of obesity in Gulf Cooperation Council (GCC) countries is more than double the global average [13], ranking them second worldwide [14]. The escalating obesity epidemic has been paralleled by a marked rise in T2D, with a reported prevalence in GCC countries ranging between 8% and 22% [15]. Similarly, among GCC countries, the interrelationship between obesity and CVD is particularly concerning, as a 1% increase in obesity prevalence is associated with an average increase of 2.7 CVD deaths per 100,000 population [16], alongside a growing incidence of stroke in the region [17]. In Qatar, recent reports showed that approximately 46% of adult women and 36% of adult men are obese [18]. The prevalence of T2D was estimated at 17% in 2021 and was projected to reach 29% by 2025 [19]. Evidently, between 50–70% of CVD cases in the Qatari population are observed among individuals with T2D [20]. CVDs continue to remain the leading cause of mortality in the country, accounting for approximately 26% of all deaths [21], with ischemic heart disease alone contributing to 21% of total mortality, thus ranking as the primary cause of death nationwide [22].

In light of the rise in obesity, T2D, and CVD, it is crucial to explore the underlying cardiometabolic risk factors contributing to these trends [23]. These include both non-modifiable elements, such as genetic predisposition, and modifiable environmental and lifestyle determinants, which often interact in complex and emergent ways to influence disease development and progression [24]. Among the key genetic contributors to cardiometabolic risk is the lipoprotein lipase (*LPL*) gene, which plays a central role in lipid metabolism by hydrolyzing circulating triacylglycerol (TG) into free fatty acids for tissue uptake [25]. In addition to its lipid-clearing function, *LPL* may exert broader protective effects through regulatory roles in glucose homeostasis, insulin sensitivity, and blood pressure control [26]. Disruption in *LPL* locus due to genetic variation has been shown to contribute to dyslipidemia and a range of metabolic phenotypes [27].

Given the critical role of *LPL* in lipid and metabolic regulation, numerous studies have investigated different *LPL* single nucleotide polymorphisms (SNPs) and their associations with adiposity [28,29], glycemic dysregulation [30,31], and dyslipidemia and cardiovascular disease risk in different populations [26,32]. Extensive research has shown that single SNP associations often explain only a small fraction of the variance in complex traits such as cardiometabolic diseases [33,34]. In contrast, aggregating multiple SNPs into a genetic risk score (GRS) captures the cumulative effect of risk alleles, offering a more robust estimate of polygenic susceptibility [35]. In this study, we examine *LPL* gene variants using a GRS approach to assess their association with obesity, T2D, and CVD risks in a healthy Qatari population. This approach enables the evaluation of polygenic risk burden in the context of a homogenous Arab Qatari cohort, offering insights into the role of *LPL* variation in shaping metabolic health in the absence of clinical disease.

## Methods

### Study population

**Fig 1:**
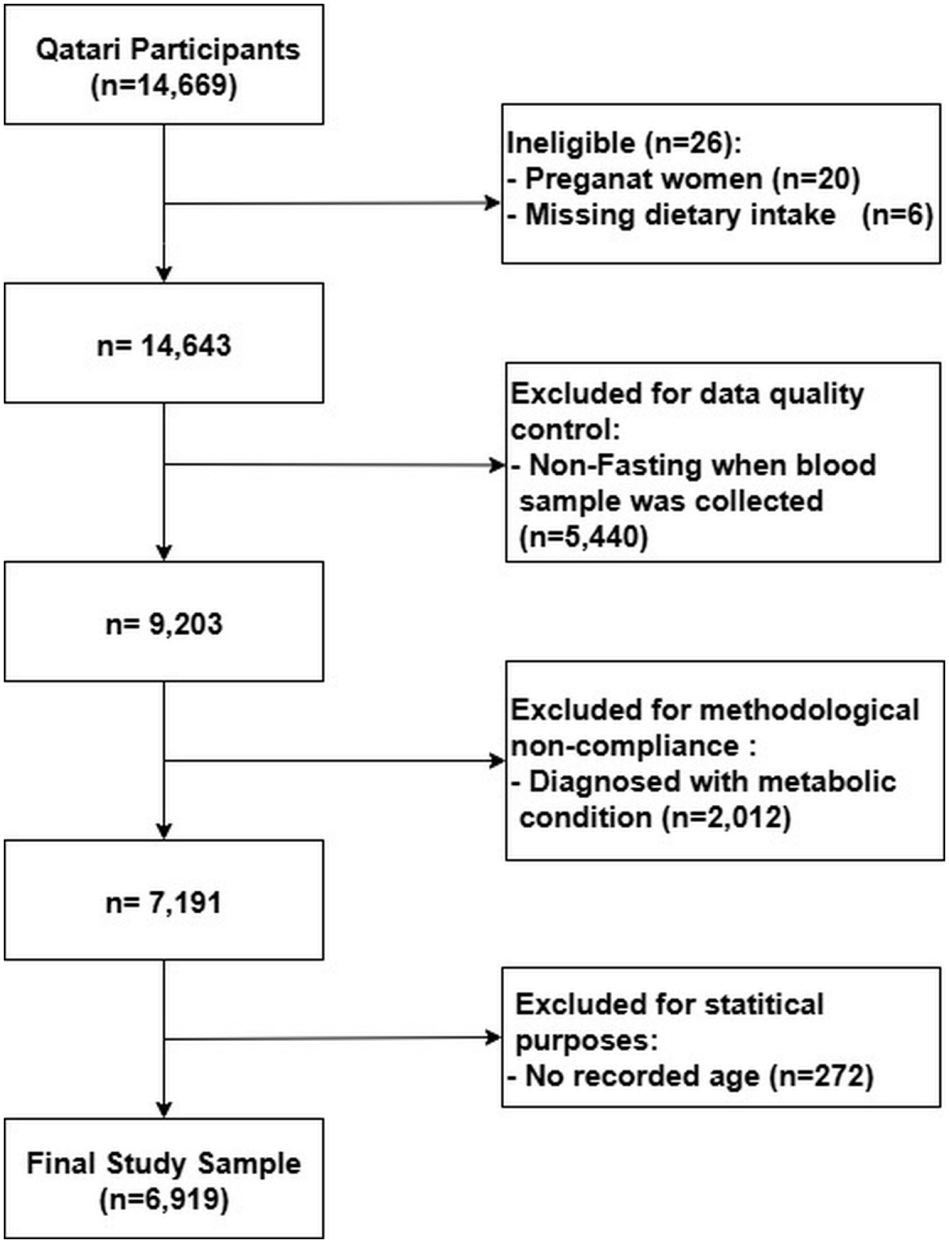
Flowchart of participant selection from the Qatar Biobank dataset. A total of 14,669 Qatari adults were initially considered. After excluding ineligible participants (n = 26; 20 pregnant women and 6 with missing dietary intake), 14,643 participants remained. Further exclusions were applied for non-fasting blood samples (n = 5,440), diagnosed metabolic conditions (n = 2,012), and missing age data (n = 272); the final analytic sample included 6,919 individuals.

The data were derived from the Qatar Biobank (QBB) Study, a population-based prospective cohort established by the Qatar Precision Health Institute (QPHI). Initiated in 2012, the QBB Study aims to recruit 60,000 participants, comprising Qatari nationals and long-term residents, following informed consent. Comprehensive details regarding the study design have been documented in prior publications [36,37]. Individuals with known metabolic disorders, such as T2D, hypertension, hypercholesterolemia, and CVDs, as well as pregnant women, were excluded from the analysis. The initial cohort comprised 14,669 Qatari participants. After excluding individuals with missing dietary intake data (n = 6) and those who reported pregnancy (n = 20), the sample was reduced to 14,643. Subsequently, participants who were not fasting at the time of blood sample collection (n = 5,440) and those diagnosed with any metabolic condition (n = 2,012) were also excluded. An additional 272 individuals were excluded due to missing age information. After applying all exclusion criteria, the final analytic sample included 6,919 metabolically healthy Qatari individuals (Fig 1).

Ethical approval was obtained in 2023 through QBB’s institutional review board (IRB: QF-QBB-RES-ACC-00085). Data access was granted in May 2022 and accessed on June 1st, 2022. Two subsequent IRB renewals were approved, extending approval until May 19th, 2025. All participant data were fully de-identified; no personal identifiers were available to the research team, as each participant was assigned a dummy ID.

### Genotyping and SNP selection

Whole-genome sequencing of Qatar Genome Program (QGP) participants was conducted using Illumina HiSeq X with 30x coverage. Standard quality control, alignment, variant calling, and annotation were performed using established pipelines (Sentieon, VEP), focusing on rare variants (<2% MAF) in 78 ACMG-recommended genes. This process, conducted by QPHI-QGP, was previously described in detail [38].

In this study, the *LPL* gene was selected due to its well-established role in lipid metabolism [27]. Variants in the *LPL* gene have been consistently associated with dyslipidemia and cardiovascular disease risks, particularly high-density lipoprotein cholesterol and TG, across different genome-wide association studies (GWAS) and meta-analysis, including rs295(C/A) [39], rs301(C/T) [39], rs320(G/T) (HindIII) [40]. Frequencies of the SNPs were obtained from the population multi-sample VCF file using BCFtools [41] and the GATK VariantsToTable tools [42]. Only *LPL* gene SNPs with a minor allele frequency (MAF) of at least 5% and at Hardy-Weinberg Equilibrium (HWE) (P ≥ 0.05) were considered for the present study.

### Cardiometabolic phenotypic markers

Phenotypic data were derived from physical and clinical assessments and categorized into the following three domains:

#### Obesity-related markers.

All anthropometric measurements were obtained using standardized protocols by trained nursing staff. Sitting and standing height, weight, and waist and hip circumferences, as well as body composition, were assessed using a Seca stadiometer and Seca Bioelectrical Impedance Analysis device (Seca GmbH & Co. KG, Hamburg, Germany). Additionally, a full-body dual-energy X-ray absorptiometry (iDXA; General Electric Company, Madison, Wisconsin) scan was performed to assess body composition [37].

The obesity indicators included were: body mass index (BMI), waist-to-hip ratio (WHR), waist circumference (WC), and fat-free mass index (FFMI).

#### Type 2 diabetes markers.

Whole blood samples were collected during clinic visits and processed at the Hamad Medical Corporation laboratories, with results returned to QBB [36]. This study focused on four glycemic control markers: glycated hemoglobin A1c (HbA1c), fasting blood glucose (FBG), fasting insulin, and fasting C-peptide.

#### Cardiovascular disease markers.

From the same set of biological samples, cardiovascular health was evaluated using key clinical markers. Blood pressure was measured in a sitting position using an Omron 705IT automated sphygmomanometer (Omron Corporation, Kyoto, Japan). Two systolic and diastolic blood pressure readings were taken at five-minute intervals, and if the measurements differed by five mmHg or more, a third reading was obtained. The average of the final readings was used for analysis [37]. In this study, six lipid profile components were included: low-density lipoprotein (LDL), high-density lipoprotein (HDL), total cholesterol (TC), and triacylglycerol (TG), systolic and diastolic blood pressure (BP).

### Statistical analysis

All statistical analyses were performed using **StataNow/SE version 18.5 for Windows (StataCorp LLC, College Station, TX, USA).** The Chi-square test for goodness-of-fit was used to assess whether the selected SNPs conformed to HWE (S1 Table). Descriptive characteristics of the study population were presented as means and standard deviations (SD) for continuous variables, and in frequencies and percentages (%) for categorical variables. Group comparisons were conducted using independent samples t-tests for continuous variables and Chi-square tests for categorical variables. The Shapiro–Wilk test was employed to assess the normality of all continuous variables including, the evaluation of skewness and kurtosis. Log transformation was applied to non-normally distributed continuous variables including, BMI, WC, FFMI, insulin, C-peptide, HbA1c, HDL, LDL, TC, TG, and SBP. Generalized Linear Models (GLM) with a Gaussian family and identity link function were used to assess associations between the *LPL*-GRS and cardiometabolic markers. Covariate adjustments were made for age, sex, and BMI (or WC when BMI was the independent variable). Post-estimation Wald tests were performed to evaluate the overall effect of GRS in each model. Bonferroni correction was applied to adjust for multiple testing (P ≤ 0.0035) based on P ≤ 0.05/ 14 outcome variables. Descriptive statistics were used to compare metabolic indicators across low- and high-GRS groups.

Risk alleles for all SNPs were determined based on their associations with lipid traits, including HDL, LDL, TG, and TC within the study population (n = 14,643). Alleles that were linked to elevated LDL, TG, or TC levels or reduced HDL concentrations were designated as risk alleles. Details on the selected risk alleles, their corresponding minor alleles, and the association results in both the study cohort and the dbSNP database are summarized in S2 Table.

An unweighted *LPL*-GRS was generated by summing the number of risk alleles across the three selected SNPs. For each SNP, individuals were assigned a score of 0, 1, or 2 corresponding to the number of risk alleles present. These individual scores were then aggregated to obtain a cumulative risk allele count. The *LPL*-GRS was subsequently divided into “low” and “high” genetic risk groups, using the median value as the cutoff point, with the “low-GRS group” comprising individuals with ≤ 5 risk alleles (n = 3,675) and the “high-GRS group” comprising individuals with >5 risk alleles (n = 3,516).

## Results

### Characteristics of participants

Participant characteristics are summarized in Table 1. Among the 6,919 individuals analyzed, 43.5% were men and 56.5% were women. When stratified by sex, BMI was significantly higher in women compared to men (p = 0.0001), with both averages falling within the overweight range. However, distinct differences emerged in body composition. Men exhibited significantly higher WC (p = 5.1x10^-190^), WHR (p = 5.1x10^-190^), and FFMI (p < 1x10^-494^). In addition, men showed significantly higher FBG, and C-peptide levels than women. More pronounced differences were seen in CVD markers, with men exhibiting higher TG, LDL cholesterol, and both systolic and diastolic BP, while women had significantly higher HDL cholesterol levels (Table 1).

**Table 1:** Clinical Characteristics of the Study Participants.

	*Total*		*Men*		*Women*		*P value*
	*Mean± SD*	*N*	*Mean± SD*	*N*	*Mean± SD*	*N*	
**Variables**							
**Age** **(years)**	34.6 ± 11.00	6,919	34.8 ± 10.8	3006	34.5 ± 11.1	3,913	0.359
**BMI** **(Kg/ m**^**2**^)	28.4 ± 6.1	6,912	28.02 ± 5.7	2,999	28.60 ± 6.4	3,913	0.0001
**WC** **(cm)**	85.3 ± 13.9	6,905	90.7 ± 13.5	2,994	81.1 ± 12.8	3,911	5.1 x10^-190^
**WHR**	0.81 ± 0.1	6,905	0.87 ± 0.1	2,994	0.67 ± 0.01	3,911	5.1x 10^–190^
**FFMI** **(kg/m**^**2**^)	17.5 ± 2.4	4,255	19.33 ± 2.04	1,610	16.38 ± 1.9	2,645	P < 1x10^-494^
**FBG** **(mmol/L)**	5.03 ± 0.88	4,252	5.12 ± 1.0	1,681	4.98 ± 0.79	2,571	6.94x10^-8^
**Fasting Insulin** **(mcunit/ mL)**	10.61 ± 7.00	4,227	11.01 ± 8.2	1,668	10.36 ± 6.1	2,559	0.0051
**Fasting C-peptide (ng/mL)**	2.01 ± 0.92	4,222	2.10 ± 1.01	1,660	1.95 ± 0.8	2,562	0.0029
**HbA1c** **(%)**	5.32 ± 0.63	6,825	5.38 ± 0.7	2,970	5.28 ± 0.6	3,855	1.14x10^-10^
**HDL** **(mmol/L)**	1.42 ± 0.51	6,892	1.23 ± 0.4	2,990	1.57 ± 0.5	3,902	2.5x10^-175^
**LDL** **(mmol/L)**	2.84 ± 0.83	6,878	3.00 ± 0.9	2,981	2.72 ± 0.8	3,897	3.4x10^-44^
**TG** **(mmol/L)**	1.28 ± 0.65	6,654	1.40 ± 0.8	2,910	1.18 ± 0.5	3,744	3.3x10^-47^
**TC** **(mmol/L)**	4.81 ± 0.91	6,898	4.86 ± 0.97	2,996	4.77 ± 0.9	3,902	0.0001
**Systolic BP** **(mmHg)**	110.00 ± 12.0	6,912	113.85 ± 11.2	3,001	107.1 ± 12.6	3,911	2.8x10^-115^
**Diastolic BP** **(mmHg)**	66.00 ± 10.0	6,912	68.98 ± 9.7	3,001	63.76 ± 8.90	3,911	1.5x10^-111^

BMI: Body Mass Index; WC: Waist Circumference; WHR: Waist-to-Hip Ratio; FFMI: Fat-Free Mass Index; FBG: Fasting Blood Glucose; HbA1c: Hemoglobin A1c; HDL: High-Density Lipoprotein; LDL: Low-Density Lipoprotein; TG: Triacylglycerol; TC: Total Cholesterol; Systolic BP: Systolic Blood Pressure; Diastolic BP: Diastolic Blood Pressure; P value: results from independent t-test**.**

### Genetic associations

#### Association of GRS with obesity-related traits.

**Fig 2:**
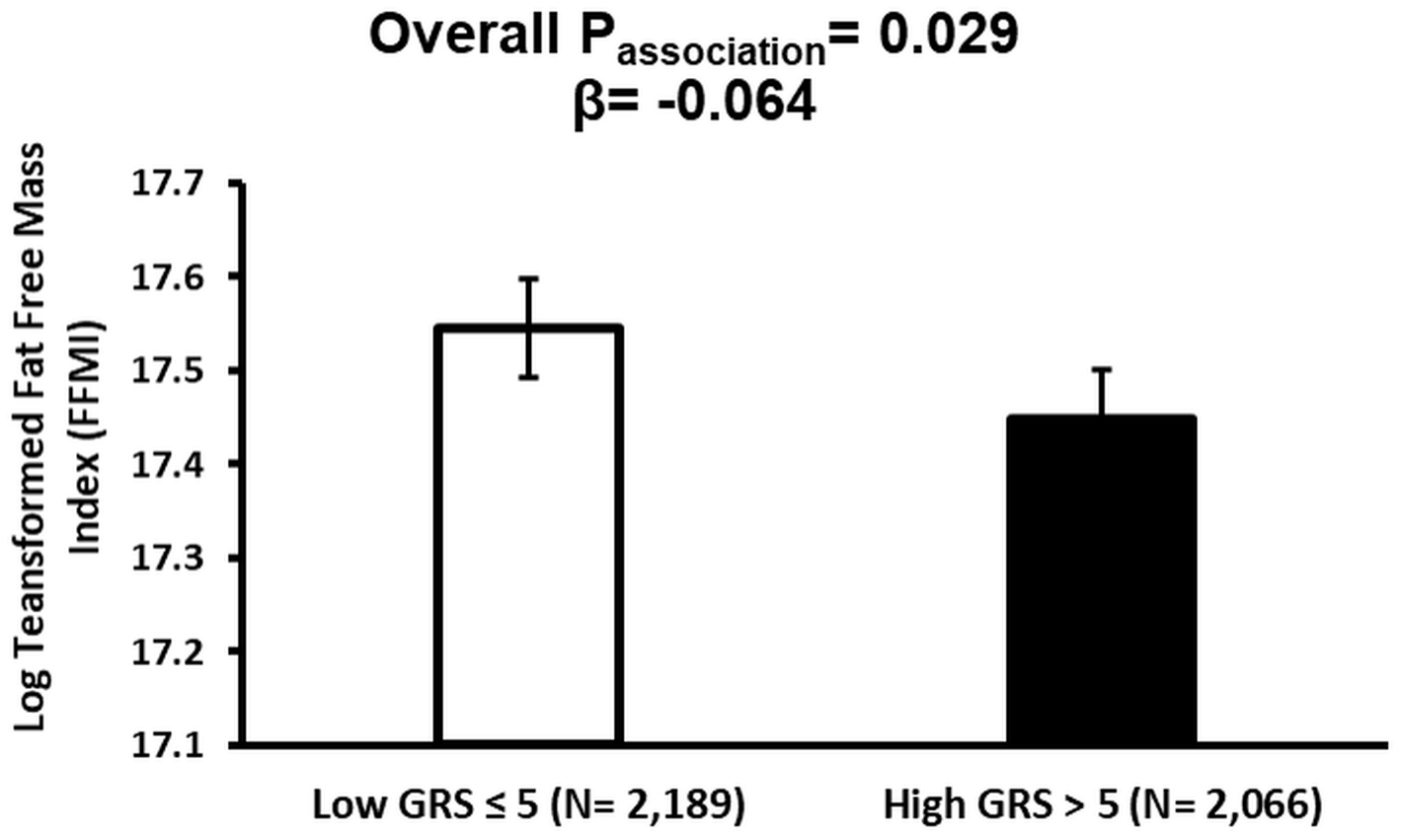
Association between *LPL*-GRS and Fat-Free Mass Index (FFMI) in the Qatar Biobank participants. The bar plot shows the adjusted mean (± SE) of log-transformed FFMI across low (≤5 risk alleles) and high (>5 risk alleles) *LPL* genetic risk score groups. The model was adjusted for age, sex, and BMI. The high GRS was significantly associated with lower FFMI (β = –0.064, p = 0.029).

The GRS was not significantly associated with BMI (p = 0.221), WC (p = 0.349), and WHR (p = 0.075). However, an association was observed between GRS and FFMI (p = 0.029), where high GRS was significantly associated with lower FFMI values (β = −0.0644, P = 0.029) (Fig 2).

Although this inverse relationship between GRS and FFMI suggests a potential genetic contribution to lean mass regulation, the association did not withstand correction for multiple testing comparisons.

#### Association of GRS with type 2 diabetes- related traits.

**Fig 3:**
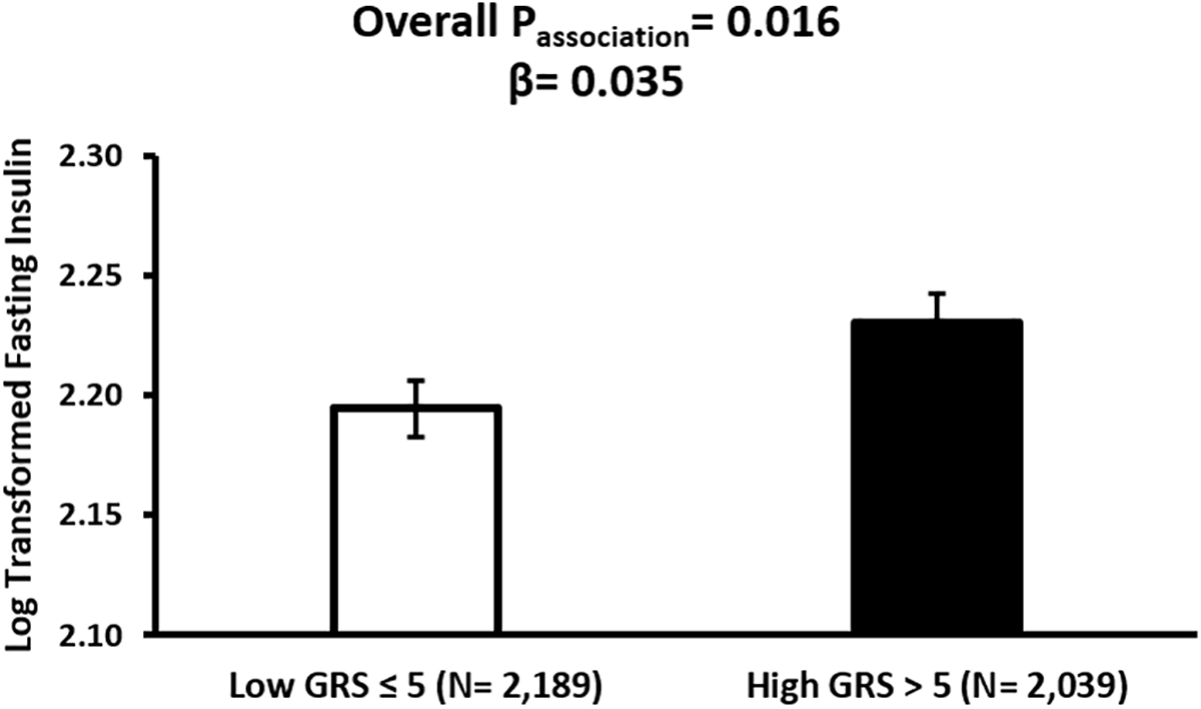
Association between *LPL*-GRS and Fasting Insulin in the Qatar Biobank participants. Adjusted mean (± SE) fasting insulin levels are shown by *LPL*-GRS category. Linear regression was adjusted for age, sex, and BMI. A positive association was observed between the high GRS and fasting insulin (β = 0.035, p = 0.016).

The fasted insulin levels were significantly associated with GRS (p = 0.016), where individuals with high GRS had significantly higher fasting insulin concentrations (β = 0.0353) (Fig 3). However, following the Bonferroni correction, this association did not retain statistical significance. While a positive trend was observed between GRS and fasting C-peptide (β = 0.0228), this association also did not reach statistical significance (p = 0.059). None of the other associations were statistically significant (FBG, p = 0.760; HbA1c, p = 0.911).

#### Cardiovascular disease associations.

**Fig 4:**
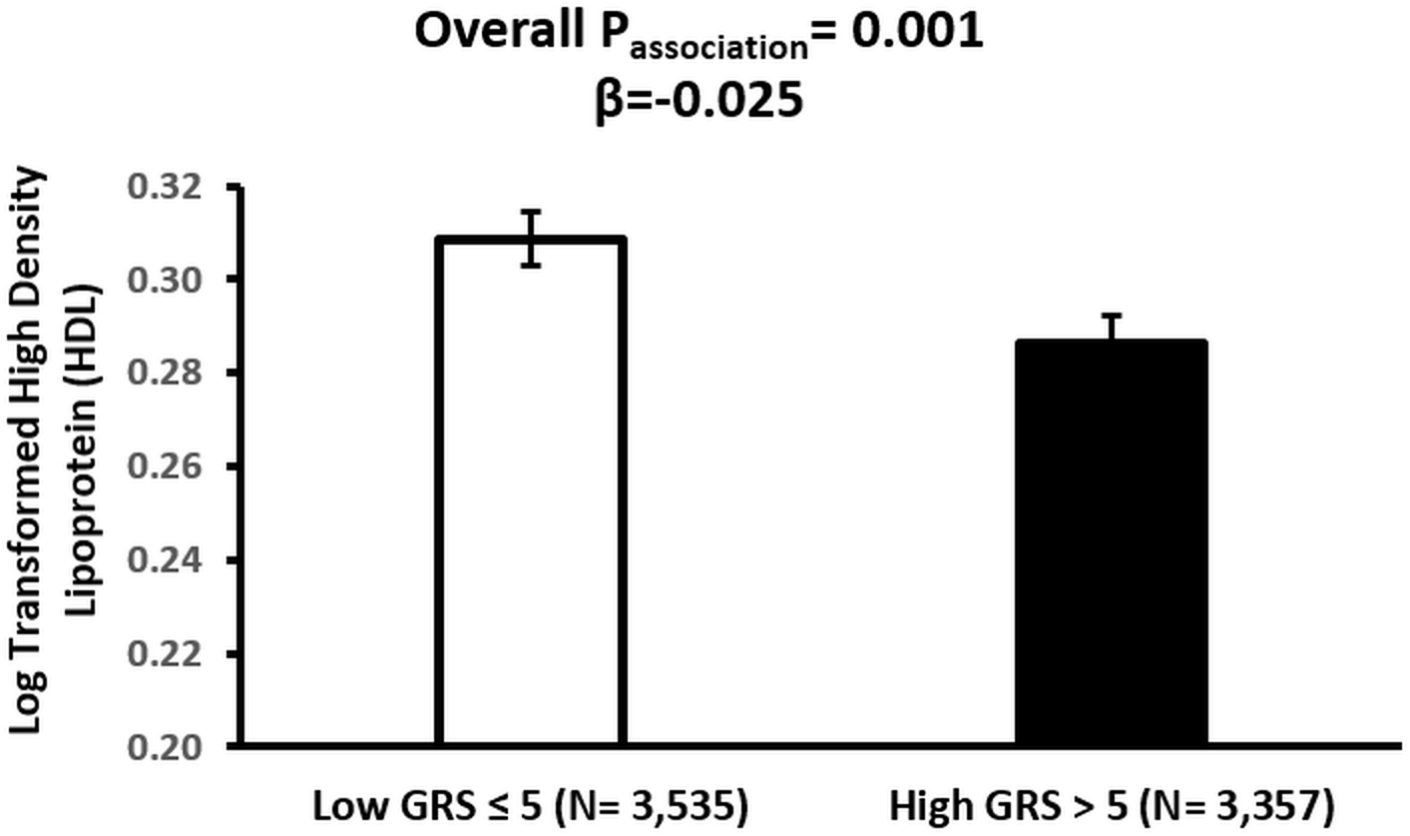
Association between *LPL*-GRS and High-Density Lipoprotein (HDL) in the Qatar Biobank participants. The bar plot displays the adjusted mean (± SE) of log-transformed HDL levels by the *LPL*-GRS group. The high GRS was significantly associated with lower HDL concentrations (β = –0.025, p = 0.001) after adjustment for age, sex, and BMI.

**Fig 5:**
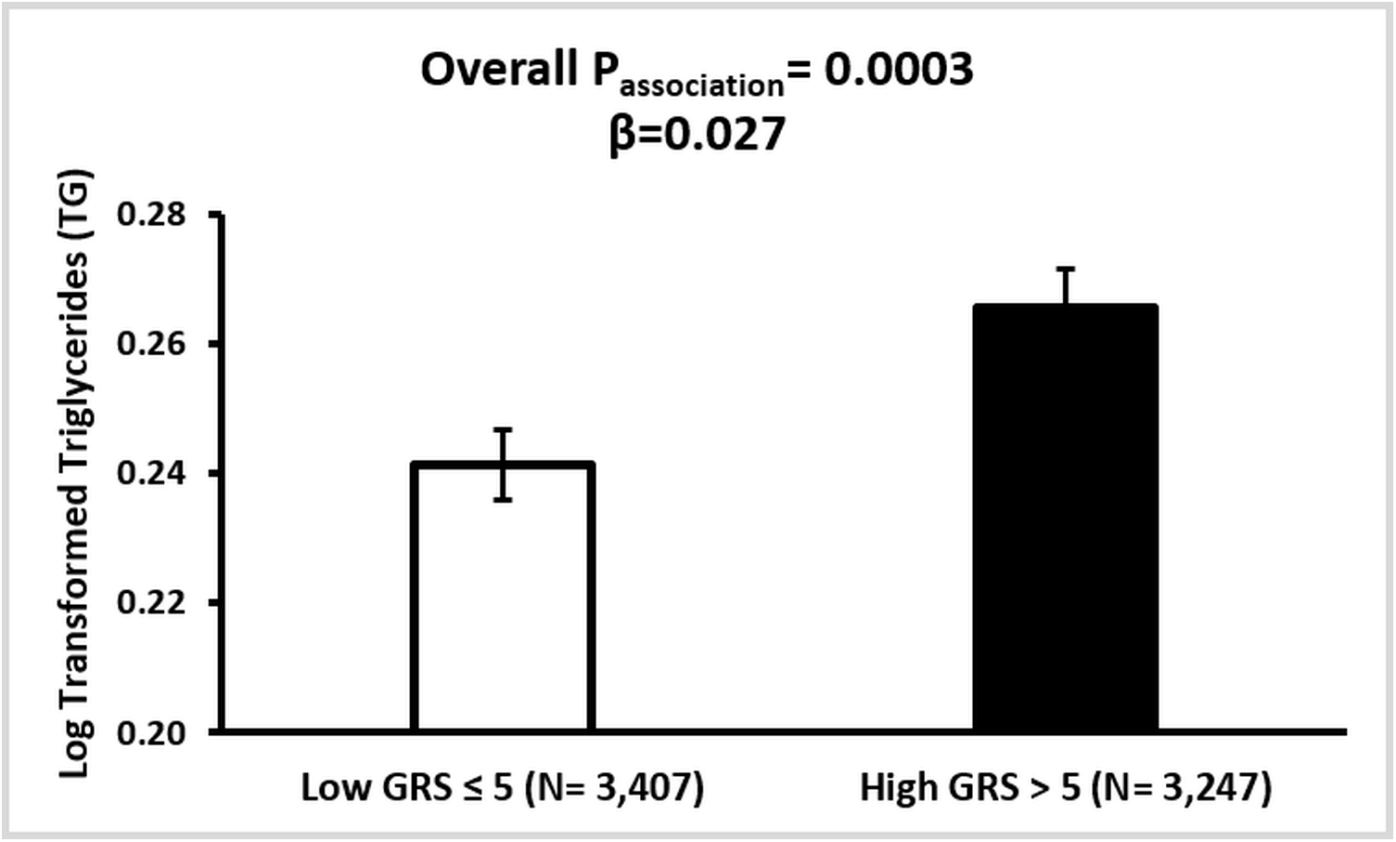
Association between *LPL*-GRS and Triacylglycerol (TG) in the Qatar Biobank participants. Adjusted (mean ± SE) log-transformed TG concentrations are presented across low and high GRS groups. The *LP*L-GRS was associated with increased log-transformed TG concentrations (β = 0.027, p = 0.0003) after adjusting for age, sex, and BMI.

**Fig 6:**
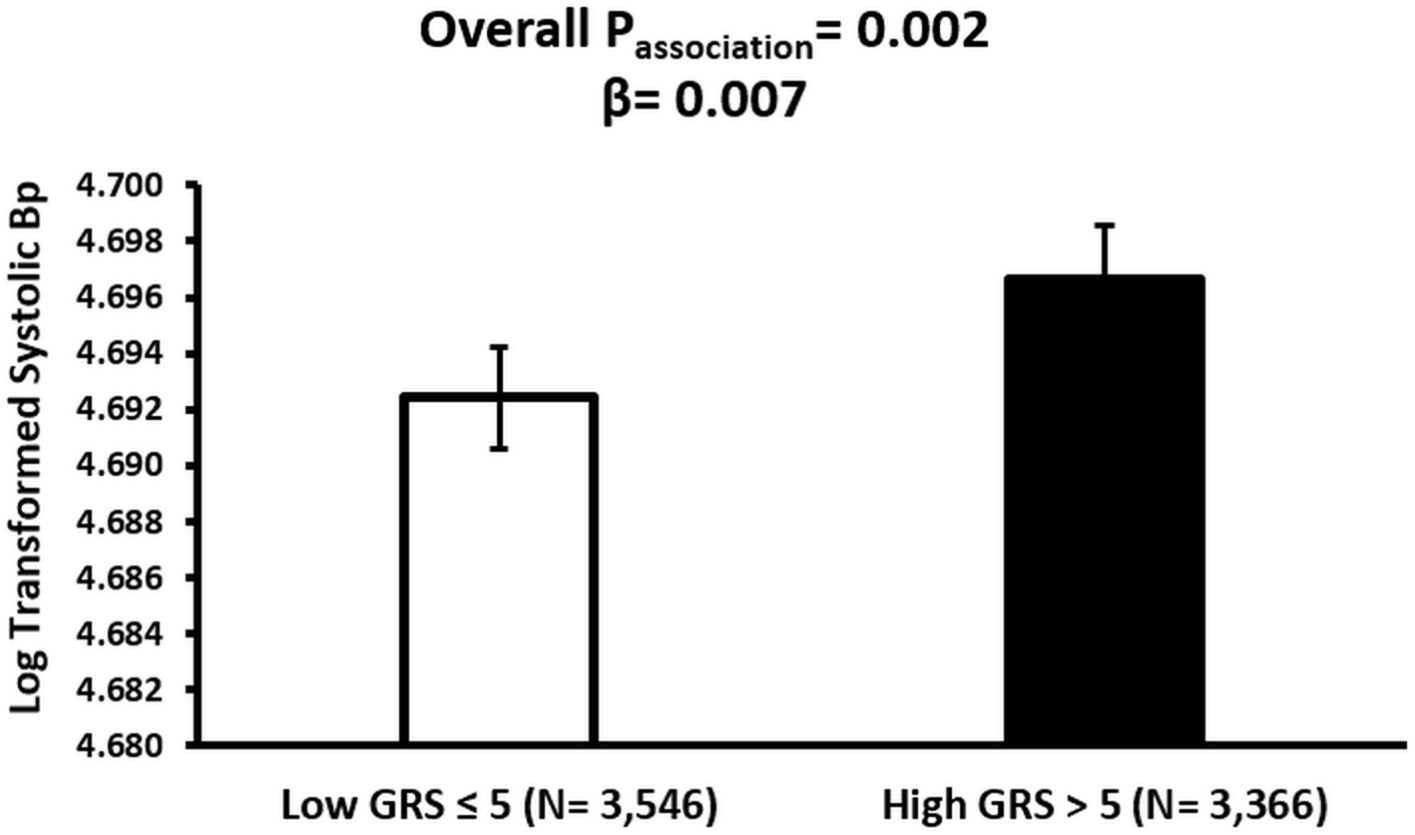
Association between *LPL*-GRS and Systolic Blood Pressure (SBP) in the Qatar Biobank participants. The bar plot presents adjusted mean (± SE) SBP by GRS category. A significant association was found between higher GRS and increased SBP (β = 0.007, p = 0.002), after adjusting for age, sex, and BMI.

The association analysis between GRS and CVD markers revealed significant findings. GRS was significantly associated with HDL levels (p = 0.001), TG concentrations (p = 0.0003), and systolic BP (p = 0.002). Individuals with high GRS exhibited lower HDL concentrations compared to those with low GRS (β = –0.0250, p = 0.001) (Fig 4). TG levels were positively associated with GRS, with significantly higher levels observed among individuals in the high GRS group (β = 0.0274, p = 0.0003) (Fig 5). Systolic BP was also significantly higher in participants with high GRS (β = 0.0067, p = 0.002) (Fig 6). The association of GRS with HDL, TG, and systolic BP remained statistically significant even after Bonferroni correction for multiple testing comparisons. No significant associations were observed between the *LPL*-GRS and LDL (β = −0.0022, p = 0.756) or TC (β = −0.0040, p = 0.352). Similarly, no significant association was detected with DBP (β = 0.0058, p = 0.071).

## Discussion

The current study investigated the association between an unweighted GRS comprising three *LPL* variants (rs295, rs301, and rs320) and various cardiometabolic risk indicators in a self-reported healthy Qatari population. The findings revealed that individuals with high *LPL*-GRS exhibited lower FFMI and HDL levels and elevated TG concentrations, systolic BP, and fasting insulin levels. These associations with CVD markers remained statistically significant after applying correction for multiple testing, underscoring the robustness of these results. These findings are particularly relevant in Qatar, where CVD is the leading cause of mortality [21]. Importantly, the risk alleles identified in our study are all common alleles in this population, which may contribute to the broader cardiometabolic disease burden. Collectively, these results demonstrate the importance of utilizing genetic association and epidemiological findings of *LPL* variants to better understand population-level risk and inform precision health efforts. Notably, clinical CAD outcomes were not directly assessed in this study; therefore, the observed associations should be interpreted as relationships with cardiometabolic risk markers linked to CAD. Consequently, the findings reflect associations with established cardiometabolic risk markers in healthy adults rather than independent prediction of CAD.

The current finding that *LPL*-GRS is associated with CVD-related markers in healthy Qatari adults is supported by prior studies on *LPL* SNPs in Arab populations. However, this is the first study to focus on a GRS using *LPL* SNPs in a Qatari population. In a Kuwaiti case-control study (n = 494), the minor “C” allele of the SNP rs295 was more common among individuals without coronary heart disease, suggesting a protective role of this single SNP against cardiovascular disease [43]. Another study in Kuwaiti adults (n = 729) found an association between the “C” allele and increased BMI but not with lipid traits [44]. The lack of association with BMI in our study may be attributed to differences in sample size and population characteristics, as our cohort consisted only of metabolically healthy individuals, whereas the Kuwaiti studies focused on comparing both healthy and diseased populations. In addition to the evidence from rs295, similar associations involving the SNP rs320 further strengthen the link between *LPL* variants and dyslipidemia across Arab populations. In a Tunisian population, individuals with the “TT” genotype of the SNP rs320 had a higher prevalence of significant coronary stenosis and nearly a threefold increased risk compared to those with the “GG” genotype. Carriers of the “T” allele also exhibited elevated TG levels and reduced HDL concentrations [45]. Similarly, in a case-control study among Iraqi individuals, the “T” allele was more common among obese participants and was associated with an increased risk of dyslipidemia [46]. Supporting evidence from Egyptian populations also links SNP rs320 to adverse metabolic outcomes, where one study (n = 100) found a significantly higher frequency of the “T” allele among diabetic individuals with dyslipidemia compared to those without [47]. Another Egyptian study (n = 200) reported that carriers of the “AT” and “TT” genotypes had higher BMI, waist circumference, fasting blood glucose, and HbA1c levels, along with reduced HDL and elevated TG, where the effects were more pronounced in diabetic participants [48]. Collectively, these findings reinforce the observed association between *LPL*-GRS and lipid abnormalities in the current study.

Evidence from large-scale non-Arab studies and meta-analyses reinforces the role of *LPL* polymorphisms in cardiometabolic health, supporting our findings in the Qatari population. A genome-wide association study of 22,161 participants across seven European cohorts identified the minor “A” allele of the SNP rs295 as a risk factor for metabolic syndrome, while the “C” allele of the SNP rs301 was linked to beneficial traits such as higher HDL and lower waist circumference [39]. A meta-analysis including 10,345 individuals from Asian and Caucasian populations also found that the minor “G” allele of rs301 was associated with a reduced risk of coronary artery disease, though this effect was limited to Caucasians [49]. In a separate meta-analysis of 89 studies (n = 22,734), the “G” allele of the SNP rs320 was associated with increased HDL (+3%), reduced TG levels (−6%), and a modest decrease in coronary heart disease risk (OR = 0.89; 95% CI: 0.81–0.98) [40]. Similarly, in Asian populations, the SNP rs320 was linked to a lower risk of ischemic stroke under both additive and dominant genetic models [50], and a review covering five studies (n = 4,996) highlighted the rs320 “G” allele as a lipid-beneficial variant associated with lower risk of metabolic syndrome and CAD [51]. Together, these findings across diverse populations validate our results and confirm the relevance of *LPL* polymorphisms in lipid metabolism. The alignment with large-scale studies further supports the robustness of our findings in the Qatari population.

The observed associations between the *LPL*-GRS and CVD markers in our Qatari cohort; together with the lack of significant associations with LDL and TC, may be explained by the physiological role of *LPL* in lipid metabolism. The LPL enzyme plays a central role in lipid metabolism by hydrolyzing TG in chylomicrons and VLDL, releasing free fatty acids for tissue uptake and contributing to HDL formation [52]. Through this upstream role in TG-rich lipoprotein catabolism, lipoprotein lipase indirectly influences the generation and composition of downstream lipoproteins; however, circulating LDL levels are primarily regulated by hepatic remodeling and LDL receptor–mediated clearance rather than by LPL-dependent lipolysis [53]. Genetic variants in *LPL* can impair enzyme function, reduce post-heparin LPL activity, and delay the clearance of TG-rich lipoproteins, resulting in elevated plasma TG [54,55]. These disruptions also hinder the supply of lipids and apolipoproteins needed for HDL synthesis and remodeling, thereby lowering HDL concentrations [55,56]. Importantly, genetic variation in LPL does not fully reflect functional lipoprotein lipase activity, which is extensively regulated at the post-translational level [55]. After synthesis in parenchymal cells, LPL requires proper folding and translocation to the vascular lumen via glycosylphosphatidylinositol-anchored high-density lipoprotein–binding protein 1 (GPIHBP1), and its activity is further modulated by circulating regulators including apolipoprotein C-II, apolipoprotein C-III, and angiopoietin-like proteins [55]. These post-translational mechanisms are highly sensitive to nutritional and hormonal states and may attenuate genotype–phenotype associations in observational studies [55]. A recent study has shown that disruptions in LPL’s ability to bind to endothelial heparan sulfate proteoglycans (HSPGs) impair its stabilization and transport to the capillary lumen, which reduces its enzymatic activity [57] and limits the availability of lipid substrates and apolipoproteins necessary for HDL particle formation and remodeling [58]. Beyond its established role in lipid metabolism, LPL also contributes to vascular function. Disrupted lipid handling at the endothelial level can result in lipid accumulation, which impairs nitric oxide production and promotes endothelial dysfunction. This process reduces vascular tone and elasticity [5]. Experimental studies further indicate that reduced LPL expression is associated with increased arterial stiffness, a key contributor to elevated systolic blood pressure [59]. In addition, LPL deficiency limits fatty acid uptake and impairs nitric oxide-mediated vasodilation, which compromises vascular responsiveness [60]. These mechanisms provide a plausible biological explanation for the observed associations between *LPL*-GRS and elevated TG, reduced HDL, and increased systolic blood pressure in our Qatari cohort. Although impaired LPL function has been linked to insulin resistance in certain populations, these associations are often modest, context-dependent, or mediated through changes in lipid metabolism, making them less consistently detectable, particularly in a healthy cohort without overt metabolic disease [61,62]. In our study, the lack of significant associations with BMI, WHR, WC, FBG, and HbA1c, as well as the failure of associations with fasting insulin and FFMI to remain significant after Bonferroni correction, reflects the greater complexity and polygenic nature of obesity and T2D. These conditions involve multiple genes and biological pathways beyond lipid metabolism, including insulin signaling, adipogenesis, inflammation, and energy balance [63–65]. Together, these findings support the interpretation that the metabolic effects of *LPL* genetic variation are likely confined to lipid-related and vascular traits rather than glycemic control or general adiposity.

The current study presents several notable strengths that enhance the robustness and relevance of its findings. First, the use of a large and well-characterized sample of 6,919 healthy Qatari adults allows for precise estimations and meaningful stratified analyses across various demographic and clinical parameters. Second, by focusing on the Arab Qatari population, the study contributes valuable data to a region underrepresented in genetic epidemiology, thereby addressing a significant gap in global health research. Third, the adoption of a GRS approach to assess the cumulative effects of three *LPL* variants provides a broader understanding of polygenic influences, as opposed to the single SNP analyses commonly used in previous studies. Finally, rigorous statistical adjustment for key covariates (age, sex, and BMI/ WC) and the application of the Bonferroni correction reduced the risk of confounding and false positives, thereby strengthening the credibility of the associations observed. Despite these strengths, several limitations must be acknowledged. Most notably, the cross-sectional design precludes causal inference and limits the ability to assess how genetic predispositions influence disease progression over time. Additionally, direct measures of enzyme activity, including post-heparin LPL activity, were not available. The unweighted nature of the GRS may also oversimplify the relative contributions of individual variants, potentially masking the impact of stronger effect alleles. Moreover, in the population-specific nature of the finding, the allele frequencies and linkage disequilibrium patterns observed may not be generalizable beyond the Qatari or broader Arab populations. Lastly, the study focused solely on three SNPs and did not explore the role of other common, rare, or structural variants in *LPL*, which may also contribute significantly to inter-individual variability in lipid metabolism and lean mass regulation. Future research addressing these limitations, particularly through longitudinal designs and inclusion of gene-environment interactions, will be essential to fully elucidate the genetic architecture of cardiometabolic risk.

## Conclusions

In summary, our study highlights that *LPL*-GRS is significantly associated with adverse cardiometabolic profiles in a healthy Qatari population. Specifically, individuals with high GRS exhibited lower HDL levels, elevated TG, and increased systolic BP, indicating increased cardiometabolic risk. However, no significant associations were observed after correction for multiple testing, with general adiposity measures and T2D markers, suggesting that the influence of these *LPL* gene variants may be more pronounced on lipid metabolism and vascular function rather than overall fat accumulation and insulin regulation. Importantly, cardiometabolic disease risk is multifactorial and reflects the combined influence of genetic susceptibility and environmental and lifestyle factors; therefore, *LPL* genetic variation represents one component of a broader cardiometabolic risk profile. Future research should focus on longitudinal studies to assess how *LPL* GRS predicts disease progression and explore gene-environment interactions with lifestyle factors. Replication in other populations and functional studies on SNP effects are also needed to support personalized prevention strategies.

## Supporting information

S1 Table Hardy Weinberg Equilibrium P-value of Lipoprotein Lipase (*LPL*) gene polymorphisms (rs295, rs301and rs320). (PDF)

S2 Table rs295, rs301, and rs320 Minor Allele Frequencies (MAF) and Risk Allele Selection. (PDF)

## Abbreviations

LPL: lipoprotein lipase
GRS: genetic risk score
SNP: single nucleotide polymorphism
QBB: qatar biobank
QPHI: qatar precision health institute
IRB: institutional review board
QGP: qatar genome program
MAF: minor allele frequency
HWE: Hardy–Weinberg equilibrium
BMI: body mass index
WC: waist circumference
WHR: waist-to-hip ratio
FFMI: fat-free mass index
HbA1c: glycated hemoglobin A1c
FBG: fasting blood glucose
TG: triacylglycerol
TC: total cholesterol
HDL: high-density lipoprotein
LDL: low-density lipoprotein
SBP: systolic blood pressure
DBP: diastolic blood pressure
GWAS: genome-wide association study
T2D: type 2 diabetes
CVD: cardiovascular disease
CAD: coronary artery disease
