# Supplementary material for: Association of lipoprotein lipase genetic risk score with cardiometabolic risk indicators in a healthy Qatari population using the Qatar Biobank data
Source: PLoS One. 2026 Feb 5;21(2):e0341641. doi: 10.1371/journal.pone.0341641 (PMC12875512; doi:10.1371/journal.pone.0341641)
Supplement: S1 Table — (PDF) [file pone.0341641.s001.pdf]

S1 Table. Hardy Weinberg Equilibrium P-value of Lipoprotein Lipase (LPL) gene polymorphisms (rs295, rs301and rs320)

No.	gene	SNP	HWE	Risk allele based on 15K	Minor allele in 15K
**1**	***LPL***	**rs301**	**0.269368682**	**T**	**C**
**2**	***LPL***	**rs295**	**0.153608882**	**A**	**C**
**3**	***LPL***	**rs320**	**0.118417664**	**T**	**G**
