# Supplementary material for: Association of lipoprotein lipase genetic risk score with cardiometabolic risk indicators in a healthy Qatari population using the Qatar Biobank data
Source: PLoS One. 2026 Feb 5;21(2):e0341641. doi: 10.1371/journal.pone.0341641 (PMC12875512; doi:10.1371/journal.pone.0341641)
Supplement: S2 Table — (PDF) [file pone.0341641.s002.pdf]

S2 Table. rs295, rs301 and rs320 Minor Allele Frequencies (MAF) and Risk Allele Selection

Gene	SNP	No.	%	HDL (mmol/L)	TG (mmol/L)	Significant Association (P-value)		Risk Allele	dpSNP MAF (Qatari)	dpSNP MAF % (sample size)	MAF (our study population)
**LPL**	**rs301**					**HDL**	**TG**				
						2.3E-06	4.9E-06	**T**	C	24.1% (52)	22.60%
Rare homozygotes	CC	783	5.3	**0.308**	0.305						
Heterozygotes	TC	5052	34.5	0.289	0.312						
Common homozygotes	TT	8808	60.2	0.265	**0.342**						
		14643									
**LPL**	**rs295**										
						4.9E-07	4.0E-06	**A**	C	25% (55)	23.70%
Rare homozygotes	CC	868	5.9	**0.305**	0.303						
Heterozygotes	AC	5214	35.6	0.29	0.313						
Common homozygotes	AA	8561	58.5	0.264	**0.342**						
		14643									
**LPL**	**rs320**										
						0.0006	0.0002	**T**	G	30.1%(65)	26%
					1.176232						
Rare homozygotes	GG	1037	7.1	**0.292**	0.306						
Heterozygotes	TG	5536	37.8	0.285	0.318						
Common homozygotes	TT	8070	55.1	0.265	**0.341**						
		14643									
